# Biological correlates before esophageal cancer screening and after diagnosis

**DOI:** 10.1038/s41598-021-96548-5

**Published:** 2021-08-23

**Authors:** Juan Zhu, Shanrui Ma, Ru Chen, Shuanghua Xie, Zhengkui Liu, Xinqing Li, Wenqiang Wei

**Affiliations:** 1 National Cancer Registry Office, National Cancer Center/National Clinical Research Center for Cancer/Cancer Hospital, Chinese Academy of Medical Sciences and Peking Union Medical College, Beijing, 100021 China; 2 Key Laboratory of Mental Health, Institute of Psychology, Chinese Academy of Sciences, Beijing, 100101 China

**Keywords:** Cancer, Cancer epidemiology, Cancer prevention, Cancer screening, Gastrointestinal cancer, Tumour biomarkers, Tumour immunology

## Abstract

Almost 50% of the world’s esophageal cancer (EC) cases occur in China, and the impact of cancer screening has long been a controversial topic. The study was designed to evaluate the biological correlates of EC screening and subsequent diagnosis in China. Based on the national cohort of esophageal cancer program, a prospective multicenter study in high-risk regions was conducted from 2017 to 2019. 61 participants received twice esophageal endoscopy screening and pathological biopsy successively (with a mean follow-up of 14.03 months). Box–Cox-power transformation and two-way repeated measures ANOVA were used to evaluate hormone cortisol and immunoglobulin (IgA, IgG, IgM) levels in plasma, reflecting their stress, immune function, and biological correlates before screening and after knowing the diagnosis. The median of cortisol, IgA, IgG, and IgM in pre-screening was 15.46 ug/dL, 1.86 g/L, 12.14 g/L, and 0.91 g/L, corresponding value at post-diagnosis was 15.30 ug/dL, 2.00 g/L, 12.79 g/L, and 0.94 g/L, respectively. No significant differences in biological indicators were found between normal and esophagitis and low-grade intraepithelial neoplasia before screening and after diagnosis. After normality transformation, cortisol, IgA, IgG and IgM levels were (0.25 ± 0.04) U/mL, (0.72 ± 0.13) (g/L), (2.44 ± 0.22) (g/L) and (0.98 ± 0.25) (g/L) before screening, (0.25 ± 0.05) U/mL, (0.70 ± 0.13) (g/L), (2.48 ± 0.21) (g/L) and (1.00 ± 0.25) (g/L) after diagnosis, respectively. Repeated Measures ANOVA showed that the main effects were significant on IgA levels between pre-screening and post-diagnosis (*P* = 0.019). No interaction effects on biological levels between pre-post screening and esophageal pathology, anxiety states (all *P* > 0.05). Little biological correlates were found both before screening and after diagnosis. Cortisol and IgA dropped less significantly, while IgM and IgA were increased slightly after diagnosis. Further multi-round longitudinal studies are needed to validate these results.

## Introduction

Esophageal cancer (EC) is one of the most common cancers in the world, and is a leading cause of cancer death, with 604,000 new cases and 544,000 deaths in 2020^1^. Nearly 50% of the world's new cases occur in China^1,2^. It may be dramatic and life-threatening, causing a considerable burden on patients, families, and society^3,4^. The impact of cancer screening has long been a controversial topic according to its benefits and disadvantages. Although several EC screening programs in high prevalence regions of China have demonstrated the efficacy of endoscopic screening in reducing the incidence and mortality of EC^5–8^, false-positive results, over-diagnosis, psychological burden, and biological change due to cancer screening are increasingly recognized^9–13^.

Growing evidence has shown that screening and diagnosis may be a stressor that stimulates participants in physical and mental ways^14–16^. The plausible biological mechanisms have been proposed that stressor stimulates and imbalances the hypothalamic–pituitary–adrenal axis (HPA), then cortisol (an essential stress hormone) is released increasingly^17–20^. Moreover, the abnormal stressor further weakens the immune function through the neuroendocrine-immune network, which plays a vital role in disease progression^15,21,22^. Objective indicators (cortisol and immunoglobulin) are more convincing compared with traditional surveys based on subjective questionnaires^23^. At present, the potential negative influence of EC screening and diagnosis is dramatically overlooked in China. Biological correlates of cancer screening and diagnosis have not been definitively identified. Evidence in this field of esophageal cancer is sparse. Therefore, the study was intended to evaluate the biological correlates of EC screening and subsequent diagnosis on cortisol and immunoglobulin levels in China and provide essential evidence and multi-facet evaluation EC screening.

## Methods

### Study design

Based on the NCEC program, a prospective, multicenter (Linzhou, Cixian) study in high-risk EC regions was conducted from 2017 to 2019^24^. All participants received endoscopy screening and pathological biopsy for free. Before screening (pre-screening), information on exposure to risk factors (e.g., smoking and alcohol drinking), blood tests (including cortisol and immunoglobulin), anxiety disorders, and physical examination were gathered uniformly. About 1 week to 1 month after the screening, screeners are notified to go to the hospital or community health center to get their screening diagnosis reports. They may be diagnosed as normal, esophagitis and LGIN, high-grade intraepithelial neoplasia (HGIN), and EC. Patients of EC and HGIN were excluded owing to the confounding effect of treatment and intervention. After knowing esophageal pathology diagnosis (post-diagnosis), the second survey was conducted to get their information about blood tests and anxiety disorders again, then track and follow-up their esophageal progression. The study flowchart is shown in Supplementary Fig. 1.

### Study participants

After an average follow-up of 14.03 months, a total of 61 residents were finally included, with the mean age of 58.57 years old (details were shown in Supplementary Table 1). The inclusion criteria were as follows: (a) aged 40–69 years old; (b) all participants signed informed consent for their voluntary participation; (c) all participants can understand questionnaire items without severe hearing or vision loss; (d) no cardia and gastric lesions. The exclusion criteria were as follows: (a) contraindications to endoscopies, such as acute perforation of the upper digestive tract, severe liver or kidney dysfunction, or heart failure; (b) Patients of EC and HGIN were excluded owing to the confounding effect of treatment and intervention; (c) Participants with diseases like Cushing syndrome, multiple myeloma, Addison's disease and other related diseases that may influence the cortisol levels or immunoglobulin abnormally.

The overall study protocol, standard operational procedure and instructions for interviews were formulated by an expert panel. Well-trained interviewers, strict standard laboratory tests, and multi-round statistical checks ensured the quality control of the study. The study was approved by the National Cancer Center/National Clinical Research Center for Cancer/Cancer Hospital, Chinese Academy of Medical Sciences and Peking Union Medical College (*No. 16-171/1250*). All procedures involving human participants were performed in accordance with the ethical committee's standards and the Helsinki declaration.

### Laboratory testing: cortisol and immunoglobulin

Plasma cortisol and immunoglobulin were tested to reflect potential biological correlates with EC screening and diagnosis. Previous studies have shown that cortisol and immunoglobulin (IgA, IgG, IgM) levels reflect their stress levels and immune function^25–27^. Peripheral venous blood was collected on an empty stomach, with a tube of 5 mL anticoagulant blood (EDTA anticoagulation blood collection tube). Gently mix upside down 6–8 times, then let it stand for about 10 min, or samples were frozen at 4 °C, then centrifuge it in a low-temperature centrifuge at 3000r/min for 10 min to separate the plasma, and process it into 0.5 mL cryopreservation tube, then samples were transported to a − 86 °C freezer.

Cortisol: automatic chemiluminescence meter MAGLUMI 2000 Plus detection instrument and cortisol determination kit were applied, which were provided by Shanghai Test Medical Laboratory. The reference range of cortisol: 5–23 ug/dL.

Immunoglobulin: IgA, IgG, IgM were detected by immunoturbidimetric method, with Toshiba automated biochemical analyzer machine (TBA-120FR) and immunoglobulin, a test kit provided by Shanghai Test Medical Laboratory. The reference range of IgA, IgG, IgM was 0.7–3.5 g/L, 7-16 g/L, 0.5–2.6 g/L, respectively.

### Questionnaire survey: generalized anxiety disorders

Generalized anxiety disorder was measured by the Chinese version of the Generalized Anxiety Disorder-7 (GAD-7, score range 0–21). GAD-7 was one of the most commonly used and acknowledged screening instruments to assess anxiety symptoms. Studies have shown the tool's good reliability and validity in primary medical care and clinical practice^28,29^. The GAD-7 includes seven items and four response scores representing the frequency of each item. (0 representing not at all, 1 representing several days, 2 representing more than half of days, and 3 representing nearly every day). The anxiety score was the total of each item. A higher anxiety score indicates worse anxiety. A score of five or greater on the GAD-7 scale represented a cut-off point for identifying anxiety symptoms^30^.

### Statistical analysis

Patients with definite basic information and complete outcomes were included for analysis. Absolute frequency and percentage were presented for categorical variables. The non-parametric Mann-Whitney U test was used due to the skewed distribution of cortisol and Immunoglobulin (IgA, IgG, IgM). Thus a Box-Cox power transformation was performed. The goal of the Box–Cox transformation is to achieve approximate normality of a variable after transformation. Roughly saying, it can be used for changing the scale of data so that the transformed data are distributed symmetrically. At the core of the Box-Cox transformation is only an exponent, lambda (λ). All values of λ are considered, and the optimal value for data is selected; the “optimal value” is the one that leads to the best approximation of a standard distribution curve^31^. Our results indicated that the optimal λ value for cortisol, IgA, IgG, IgM was − 0.50, − 0.50, 0, 0.36, respectively (Supplementary Fig. 2).

Furthermore, repeated-measures analysis of variance (two-way ANOVA) was used to compare cortisol, IgA, IgG and IgM levels between pre-screening and post-diagnosis. Subgroup analyses were performed by esophageal pathology and anxiety states. Data management, programming, and analyses were conducted using Minitab 19 (Minitab, State College, PA) and SAS 9.4 (SAS Institute Inc., Cary, NC, USA). All tests of significance were two-tailed, and *P* < 0.05 was considered statistically significant.

## Results

### Cortisol and immunoglobulin levels among participants with different esophageal pathology grades

As shown in Table 1, the median (Q1–Q3) of cortisol, IgA, IgG and IgM at pre-screening were 15.46 (11.92–18.73) ug/dL, 1.86 (1.48–2.61) g/L, 12.14 (9.61–13.81) g/L and 0.91 (0.67–1.33) g/L respectively. After participants knowing the first screening diagnosis results (at post-diagnosis), the median (Q1–Q3) of cortisol, IgA, IgG, and IgM were 15.30 (11.68–21.23) ug/dL, 2.00 (1.65–2.85) g/L, 12.79 (10.02–14.15) g/L and 0.94 (0.68–1.27) g/L, respectively. Regardless of whether before screening or after knowing the diagnosis, there were no significant differences in cortisol, IgA, IgG, and IgM levels between normal and esophagitis and LGIN groups (all *P* > 0.05).

**Table 1:** Cortisol and immunoglobulin levels among participants with different esophageal pathology grades.

	Esophageal pathology	Pre-screening		Post-diagnosis	
		Median (Q1–Q3)	P*	Median (Q1-Q3)	P*
Cortisol (ug/dL)	Total	15.46 (11.92–18.73)	0.161	15.30 (11.68–21.23)	0.259
	Normal and esophagitis	16.63 (11.75–23.65)		16.50 (11.92–22.48)	
	LGIN	13.98 (12.27–16.34)		14.09 (11.53–20.14)	
IgA (g/L)	Total	1.86 (1.48–2.61)	0.681	2.00 (1.65–2.85)	0.983
	Normal and esophagitis	1.88 (1.58–2.59)		2.06 (1.54–2.83)	
	LGIN	1.79 (1.46–2.65)		1.94 (1.65–2.92)	
IgG (g/L)	Total	12.14 (9.61–13.81)	0.121	12.79 (10.02–14.15)	0.516
	Normal and esophagitis	12.51 (10.03–14.03)		12.55 (9.98–14.84)	
	LGIN	10.54 (8.88–13.59)		12.93 (9.98–14.05)	
IgM (g/L)	Total	0.91 (0.67–1.33)	0.296	0.94 (0.68–1.27)	0.282
	Normal and esophagitis	1.02 (0.67–1.37)		1.07 (0.67–1.48)	
	LGIN	0.83 (0.68–1.17)		0.90 (0.67–1.16)	

Esophageal pathology: Normal (n = 23), Esophagitis (n = 8), LGIN (n = 30).

*Q1* lower quartile (25%), *Q3* upper quartile (75%), *LGIN* Low-grade intraepithelial neoplasia.

*Mann–Whitney U test.

### Comparison of cortisol and immunoglobulin levels among participants at pre-screening and post-diagnosis: by esophageal pathology grades

**Figure 1:**
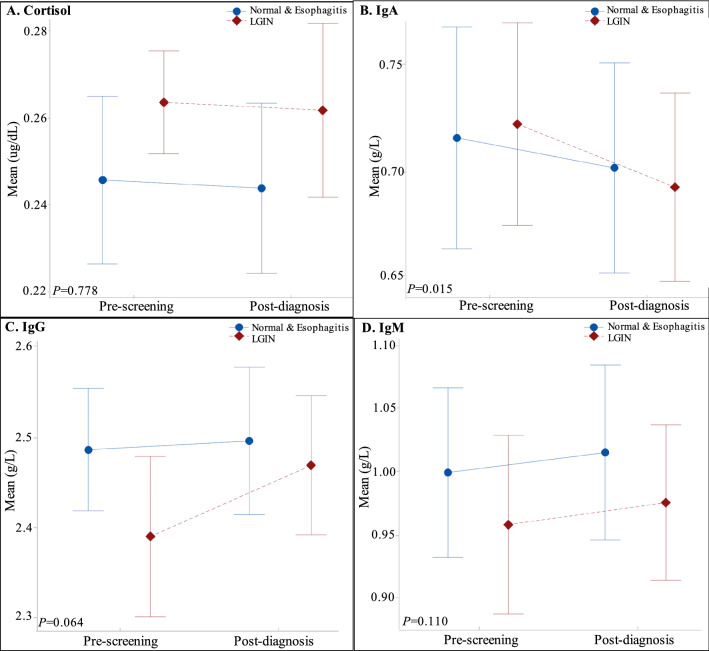
Comparison of cortisol and immunoglobulin levels among participates at pre-screening and post-diagnosis: by esophageal pathology grades. (**A**) Mean values of cortisol; (**B**) Mean values of IgA; (**C**) Mean values of IgG; (**D**) Mean values of IgM.

After Box-Cox normality transformation, cortisol, IgA, IgG and IgM levels were (0.25 ± 0.04) U/mL, (0.72 ± 0.13) (g/L), (2.44 ± 0.22) (g/L) and (0.98 ± 0.25) (g/L) before screening, and the corresponding levels were (0.25 ± 0.05) U/mL, (0.70 ± 0.13) (g/L), (2.48 ± 0.21) (g/L) and (1.00 ± 0.25) (g/L) after knowing diagnosis, respectively. A slight dip in cortisol and IgA and a small increase in IgM and IgA after a period of diagnosis. Repeated measures ANOVA showed that the main effects were significant on IgA levels between pre-screening and post-diagnosis (*P* = 0.015). There were no significant differences in cortisol, IgG, and IgM levels in pre-screening and post-diagnosis (*P* = 0.778, *P* = 0.064, *P* = 0.110). No interaction effects on biological levels between screening and esophageal pathology (all *P* > 0.05). Details are shown in Table 2 and Fig. 1.

**Table 2:** Comparison of cortisol and immunoglobulin levels among participants at pre-screening and post-diagnosis: by esophageal pathology grades.

	Pre-screening (Mean ± SD)			Post-diagnosis (Mean ± SD)			Repeated measures ANOVA	
	Normal and esophagitis (n = 31)	LGIN (n = 30)	Total (n = 61)	Normal and esophagitis (n = 31)	LGIN (n = 30)	Total (n = 61)	P*	P**
Cortisol (ug/dL)	0.25 ± 0.05	0.26 ± 0.03	0.25 ± 0.04	0.24 ± 0.05	0.26 ± 0.05	0.25 ± 0.05	0.778	0.996
IgA(g/L)	0.72 ± 0.14	0.72 ± 0.13	0.72 ± 0.13	0.70 ± 0.13	0.69 ± 0.12	0.70 ± 0.13	0.015	0.378
IgG(g/L)	2.49 ± 0.18	2.40 ± 0.24	2.44 ± 0.22	2.50 ± 0.22	2.46 ± 0.21	2.48 ± 0.21	0.064	0.146
IgM(g/L)	0.10 ± 0.18	0.96 ± 0.19	0.98 ± 0.25	1.02 ± 0.19	0.98 ± 0.16	1.00 ± 0.25	0.110	0.939

Data did not show normal distributions were transformed using Box-Cox-power transformation. All values of λ are considered the optimal value for data is selected; the “optimal value” is the one that results in the best approximation of a normal distribution curve.

*LGIN* Low-grade intraepithelial neoplasia.

*Main effect.

**Interaction with pathology.

### Comparison of cortisol and immunoglobulin levels among participants at pre-screening and post-diagnosis: by anxiety disorders status

**Figure 2:**
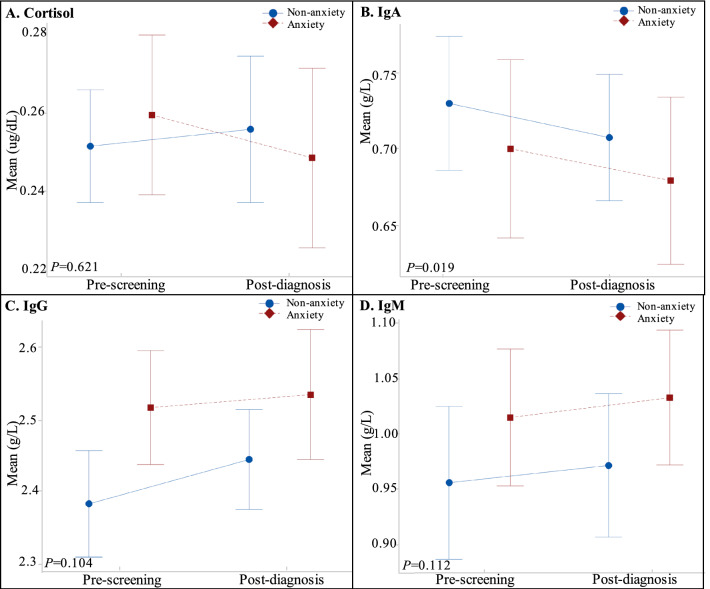
Comparison of cortisol and immunoglobulin levels among participates at pre-screening and post-diagnosis: by anxiety disorders status. (**A**) Mean values of cortisol; (**B**) Mean values of IgA; (**C**) Mean values of IgG; (**D**) Mean values of IgM.

Before the screening, the anxiety level was significantly higher than that after diagnosis (40.98% vs. 18.03%, *P* < 0.001). The repeated measures ANOVA showed that the main effects were significant on IgA levels at pre-screening and post-diagnosis (*P* = 0.019). No significant main effects on cortisol, IgG, and IgM levels were observed at pre-screening and post-diagnosis (*P* = 0.624, *P* = 0.104, *P* = 0.112). Besides, no interaction effects between screening and anxiety states (all *P* > 0.05). Details are presented in Table 3 and Fig. 2.

**Table 3:** Comparison of cortisol and immunoglobulin levels among participants at pre-screening and post-diagnosis: by anxiety disorders status.

	Pre-screening (Mean ± SD)			Post-diagnosis (Mean ± SD)			Repeated measures ANOVA	
	Normal (n = 36)	Anxiety (n = 25)	Total (n = 61)	Normal (n = 36)	Anxiety (n = 25)	Total (n = 61)	P*	P**
Cortisol (ug/dL)	0.25 ± 0.04	0.26 ± 0.05	0.25 ± 0.04	0.26 ± 0.05	0.25 ± 0.05	0.25 ± 0.05	0.624	0.262
IgA(g/L)	0.73 ± 0.13	0.70 ± 0.14	0.72 ± 0.13	0.71 ± 0.12	0.68 ± 0.13	0.70 ± 0.13	0.019	0.932
IgG(g/L)	2.38 ± 0.22	2.52 ± 0.19	2.44 ± 0.22	2.44 ± 0.20	2.53 ± 0.22	2.48 ± 0.21	0.104	0.371
IgM(g/L)	0.96 ± 0.20	1.01 ± 0.15	0.98 ± 0.25	0.97 ± 0.19	1.03 ± 0.15	1.00 ± 0.25	0.112	0.915

Data did not show normal distributions were transformed using Box-Cox-power transformation. All values of λ are considered the optimal value for data is selected; the “optimal value” is the one that results in the best approximation of a normal distribution curve.

*LGIN* Low-grade intraepithelial neoplasia.

*Main effect.

**Interaction with pathology.

## Discussion

The present study provides innovative evidence on biological correlates of cancer screening and diagnosis, which partly fill the gap in how the screening and diagnosis (psychological distress) affects hormone levels and immune function. In the study, little biological correlate was found both before screening and after diagnosis. After knowing the screening diagnosis, cortisol and IgA levels showed a slightly decreasing trend. The findings suggested that the potential adverse impact of cancer screening may exist, which may provide a scientific reference for optimizing the multi-facet evaluation of cancer screening strategy.

As the essential glucocorticoid and steroid hormone, cortisol is acknowledged as a sensitive indicator of the stress response^18–20,25^. Previous evidence indicated that endoscopic screening played a crucial role in participants’ psychological health, especially increasing their stress and anxiety disorders. The possible explanation was that cancer-related diagnosis in screening might act as a severe stressor and stimulation of life-stress events, especially for patients screened as HGIN and EC. They were susceptible to fall into a gloomy, depressed, and painful mood^13–15^. The plausible biological mechanisms have been proposed that stressor stimulates and imbalances the hypothalamic–pituitary–adrenal axis (HPA). The work of stress is closely related to the pituitary gland and adrenal gland. HPA is activated and releases corticotrophin-releasing hormone (CRH) and adrenocorticotropic hormone (ACTH) and then increases the secretion of glucocorticoid. Then, the adrenal gland released cortisol hormone into the blood increasingly and circulated it throughout the body^17–20^. Abnormal cortisol levels may result in an allosteric load to our body, leading to metabolic disorders in the body’s regulatory networks^32^. Accordingly, the variation of cortisol could be taken as the physiological and biological response to stress to some extent^25,26,33,34^. The stress response is essentially a self-protection mechanism when our body is threatened by internal and external environmental factors, social and psychological factors. As a vital stress indicator, cortisol can be considered to measure the effectiveness of stress interventions in future studies.

The study found no significant difference in cortisol and immunoglobulin levels among participants with different esophageal pathology grades, regardless of pre-screening or post-diagnosis. One plausible explanation may be that the study was based on the general population in ECHRRs, and their health status generally seems to be at the same horizon level. The recruited screeners appear to be asymptomatic healthy, most of which were diagnosed as normal, esophagitis, and LGIN. Before the screening, participants do not know what the screening diagnosis would be afterward, which can be considered as no stimulus^14^. After knowing the screening diagnosis, cortisol levels in both normal and esophagitis and LGIN pathological grade have a slightly decreased trend.

Furthermore, we found that cortisol levels among participants with LGIN were higher than normal participants. The main reason was that LGIN diagnosis might be a stressful life event and a potential stimulus, especially for precancerous lesions. When high-risk states are screened, the patient may be hard to accept for a short time and may get distressed about cancer progression, treatment, recurrence, and metastasis^13^. The precancerous diagnosis activated the HPA, secreted cortisol excessively, and weakened the body’s immune function through the neuro-endocrine-immune network abnormally^35,36^. When low-risk health states are screened (e.g., esophagitis), regular follow-up and habits and customs change are recommended, which will reduce psychological stress and fear of disease among participants so that the cortisol level will be slightly reduced^37^.

Immunoglobulin (Ig) plays a vital role in the immune system, with antibody function and specificity, synthesized and secreted when external pathogens or other substances activate the human immune cells. Previous studies showed that Ig levels might be linked to tumorigenesis and progression^38,39^. With the progress of malignant tumors, patients' immunity appears on a downward trend^40^. In our study, compared with before screening, IgA increased significantly after diagnosis. Moreover, IgA, IgG, and IgM levels in the LGIN group was slightly lower than in the normal and esophagitis group, which indicated that a worse immune dysfunction in the LGIN group after knowing the screening diagnosis, which indicated that screening and diagnosis were potentially detrimental to participants by invading the neuroendocrine-immune function through the hypothalamic–pituitary–adrenal axis (HPA). These patterns are similar to other types of cancer in previous studies^41,42^. The possible reason is that the occurrence of precancerous lesions may inhibit Ts cells and weaken the ability to control B cell’s differentiation; thereby less Ig antibodies were synthesized and secreted in serum. Our results showed that IgA levels declined obviously among anxious participants, which may be explained that chronic stress may induce immuno-regulatory suppression mechanisms, thereby reducing the numbers of regulatory T-cells and B-cells (Tregs and Bregs), and suppress anti-tumor immune responses after knowing the pathological results^43,44^. Participants with anxiety showed higher IgG and IgM levels than normal people. The possible mechanism may be linked to that negative emotions and stressful events may promote proinflammatory cytokines (such as IL-6, IL-1β, and C-Reactive Protein) that influence chronic inflammation proinflammatory and autoimmune disorders^45^.

Cancer diagnosis can profoundly affect psychological state and cortisol rhythms^46^. Cortisol has received increasing attention due to its vital role in mental disorders such as anxiety and depression. Several studies indicated that the plasma cortisol level of patients with anxiety or depression is higher than normal people^47–50^. An explanation was that mental disorders stimulate the central nervous system, activated the HPA, and released cortisol. Nevertheless, our results showed that no discernible relationship was observed between cortisol levels and generalized anxiety disorders, which were in line with earlier research^51,52^.

The psychological burden of screening can not be ignored and overlooked^10,11,53^. It is reflected in the following aspects: (1) extra treatment owing to false-positive diagnosis, causing economic, social and patients' physical and mental burden^9,54^; (2) over-diagnosis bring about unnecessary diagnosis and treatment afterward and considerate psychological stress, and borderline lesions without clinical symptoms are largely unchanged in their lifetime)^12,55,56^; (3) As several countries in the Middle East or Asia, diagnosis of cancer is regarded as equivalent to death. Family members may ask doctors not to tell patients about the diagnosis or the word "cancer"^57^. The mental pressure of informing screening diagnosis (EC and precancerous lesions) is lasting and far-reaching. Therefore, controlling and alleviating stress sources may be beneficial to improve the HPA imbalance and immune dysfunction, which may be a breakthrough in simplifying screening strategies and concentrating on high-risk populations.

Both biological measures (cortisol and immunoglobulin) and questionnaire interviews were conducted in our follow-up study, mainly avoiding the subjective deviation. The outcome was all confirmed by pathological diagnosis (gold standard). Several limitations should be acknowledged. First, the sample size is not large enough, but our exploratory study largely provides clues and references from a novel perspective and fills the gap in this field. Second, causal inferences cannot be determined, and further research on mechanisms is needed. Third, potential selection and information bias may exist, and unmeasured and residual confounding may be detrimental to the results' interpretation. Fourth, the secretion of cortisol is rhythmic, and we tested participants' blood in the morning, which may cause the results to show no difference. Finally, screeners' results may not be generalized to the general population and should be interpreted cautiously.

## Conclusions

In this prospective study, with biological measures and questionnaire interviews, we found little biological correlation before screening and after diagnosis. Cortisol and IgA dropped less significantly, while IgM and IgA were increased slightly after diagnosis. The findings suggested that the potential adverse impact of cancer screening may exist, which may provide a scientific reference for optimizing the multi-facet evaluation of cancer screening strategy. Further multi-round longitudinal studies are required to validate these results.

## Supplementary Information

Supplementary Legends.

Supplementary Figure 1.

Supplementary Figure 2.

Supplementary Table 1.
