# Supplementary material for: Biological correlates before esophageal cancer screening and after diagnosis
Source: Sci Rep. 2021 Aug 23;11:17015. doi: 10.1038/s41598-021-96548-5 (PMC8382699; doi:10.1038/s41598-021-96548-5)
Supplement: Supplementary file 1 — Supplementary Legends. [file 41598_2021_96548_MOESM1_ESM.docx]

**Supplementary Legends**

**Supplementary Figure 1.** The flowchart of study

**Supplementary Figure 2.** Box-Cox plot and the optimal value λ of cortisol, IgA, IgG and IgM

**Supplementary Table 1.** Basic characteristics of participants
