# Supplementary figures and images for: Biological correlates before esophageal cancer screening and after diagnosis
Source: Sci Rep. 2021 Aug 23;11:17015. doi: 10.1038/s41598-021-96548-5 (PMC8382699; doi:10.1038/s41598-021-96548-5)

A

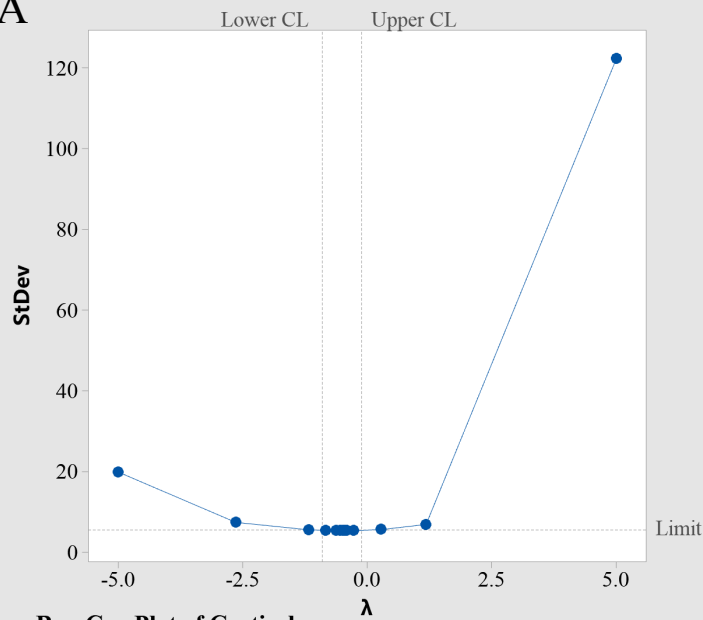

B

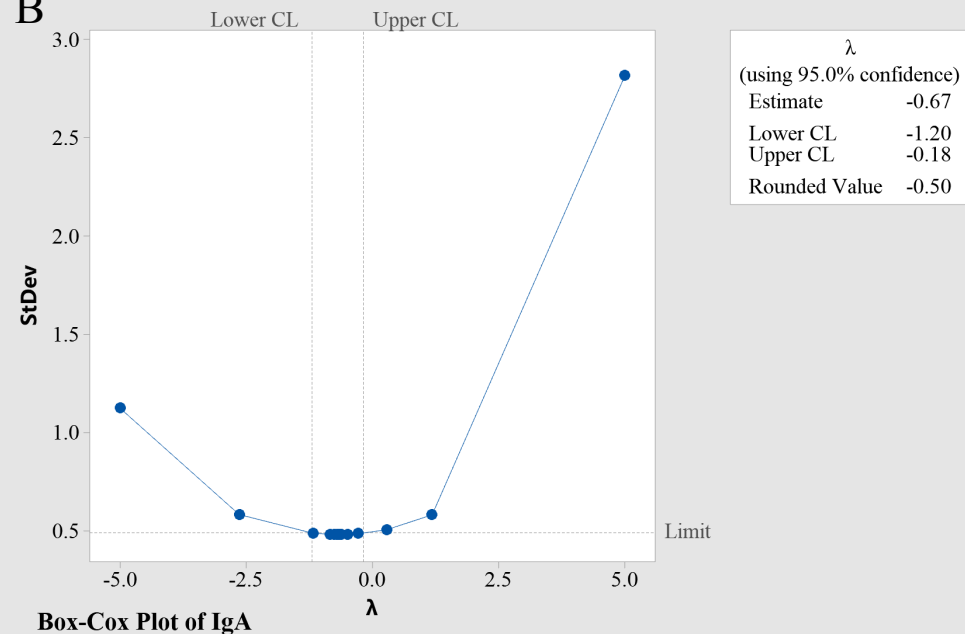

C

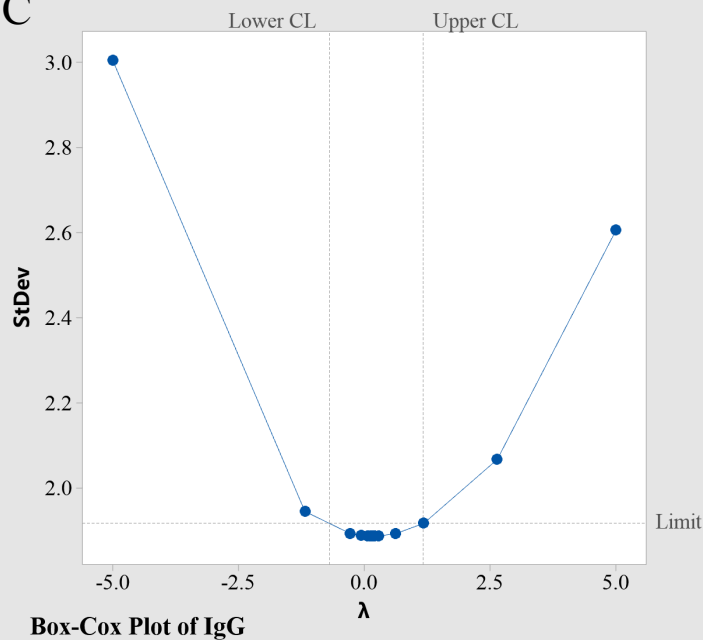

D

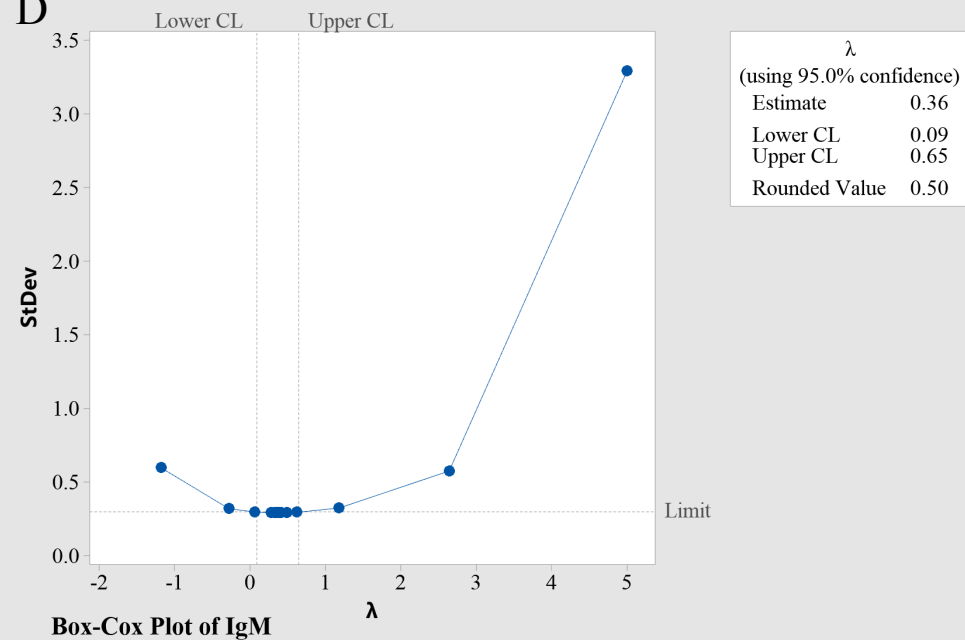

**Supplementary Figure 2** Box-Cox plot and the optimal value  $\lambda$  of cortisol, IgA, IgG and IgM

Supplement: Supplementary file 3 — Supplementary Figure 2. [file 41598_2021_96548_MOESM3_ESM.pdf]
