# Supplementary material for: Biological correlates before esophageal cancer screening and after diagnosis
Source: Sci Rep. 2021 Aug 23;11:17015. doi: 10.1038/s41598-021-96548-5 (PMC8382699; doi:10.1038/s41598-021-96548-5)
Supplement: Supplementary file 4 — Supplementary Table 1. [file 41598_2021_96548_MOESM4_ESM.doc]

| **Supplementary Table 1** Basic characteristics of participants | | |
| --- | --- | --- |
| Characteristics | N | % |
| Age at survey, year (Mean±SD) | 58.57 ± 7.46 | |
| Follow-up time, month (Mean±SD) | 14.03 ± 9.56 | |
| High-risk region |  |  |
| Linzhou | 41 | 67.21 |
| Cixian | 20 | 32.79 |
| Gender |  |  |
| Male | 29 | 47.54 |
| Female | 32 | 52.46 |
| Marital status |  |  |
| Married | 58 | 95.08 |
| Widowed | 3 | 4.92 |
| Highest education level |  |  |
| Primary school or below | 38 | 62.30 |
| High school or Colleges | 23 | 37.70 |
| Occupation |  |  |
| Agriculture and related workers | 46 | 75.41 |
| Factory workers, technical, sales or service workers | 2 | 3.28 |
| Housewife or househusband | 11 | 18.03 |
| Private owners or other | 2 | 3.29 |
| Household income, ten thousand RMB/year |  |  |
| <3.0 | 13 | 21.31 |
| 3.0-7.0 | 37 | 60.66 |
| 7.0-11.0 | 7 | 11.48 |
| ≥11.0 | 2 | 3.28 |
| Unknown | 2 | 3.28 |
| Smoking frequency |  |  |
| Do not smoke | 50 | 81.97 |
| Only occasionally | 2 | 3.28 |
| Most days or almost everyday | 9 | 14.75 |
| Alcohol consumption frequency |  |  |
| Do not drink alcohol | 49 | 80.33 |
| Only occasionally | 10 | 16.39 |
| Most days or almost everyday | 2 | 3.28 |
| Physical exercise |  |  |
| <1-3 times/month | 55 | 90.16 |
| 3-5 times/week or almost everyday | 6 | 9.84 |
| Life satisfaction |  |  |
| Very satisfied | 10 | 16.39 |
| Basically satisfied | 50 | 81.97 |
| General | 1 | 1.64 |
| Self-rated health status |  |  |
| Excellent | 9 | 14.75 |
| Good | 25 | 40.98 |
| Fair-poor | 27 | 44.26 |
| Hypertension |  |  |
| Yes | 13 | 21.31 |
| No | 32 | 52.46 |
| Unknown | 16 | 26.23 |
| Diabetes |  |  |
| Yes | 3 | 4.92 |
| No | 42 | 68.85 |
| Unknown | 16 | 26.23 |
| Anxiety |  |  |
| Yes | 36 | 59.02 |
| No | 25 | 40.98 |
| Esophageal pathology (Endoscopy screening) |  |  |
| Normal | 23 | 37.70 |
| Esophagitis | 8 | 13.11 |
| LGIN | 30 | 49.18 |
| LGIN = Low-grade intraepithelial neoplasia | | |
